# Low responders may benefit from undergoing ovarian stimulation with a long GnRH agonist protocol with corifollitropin alfa followed by hMG

**DOI:** 10.5935/1518-0557.20230001

**Published:** 2023

**Authors:** Ana Filipa Ferreira, Ana Sofia Pais, Ana Paula Sousa, Paulo Cortesão, Teresa Almeida-Santos

**Affiliations:** 1 Reproductive Medicine Unit, Gynecology, Obstetrics, Reproduction and Neonatology Department, Centro Hospitalar e Universitário de Coimbra, Praceta, Coimbra, Portugal; 2 University of Coimbra, Faculty of Medicine, Azinhaga de Santa Comba, Celas, Coimbra, Portugal; 3 CNC-Center for Neuroscience and Cell Biology, Center for Innovative Biomedicine and Biotechnology (CIBB), Azinhaga de Santa Comba, Celas, University of Coimbra, Portugal; 4 University of Coimbra, Coimbra Institute for Clinical and Biomedical Research (iCBR) Area of Environment Genetics and Oncobiology (CIMAGO), Biophysics Institute of Faculty of Medicine, Coimbra, Portugal; 5 University of Coimbra, Center for Innovative Biomedicine and Biotechnology (CIBB), Coimbra, Portugal; 6 Clinical Academic Center of Coimbra (CACC), Coimbra, Portugal

**Keywords:** ovarian stimulation protocol, low response, long GnRH-agonist protocol, corifollitropin alfa, hMG

## Abstract

**Objective:**

To evaluate the outcomes of a long GnRH agonist protocol with corifollitropin alfa followed by hMG in low responders.

**Methods:**

Retrospective cohort study. Patients with a suboptimal previous ovarian response (<9 oocytes) and a normal ovarian reserve (Poseidon groups 1 and 2) were classified in 1) Group 1 (n=88), submitted to a second cycle with a GnRH antagonist protocol using rFSH/hMG; 2) Group 2 (n=66), submitted to a long GnRH agonist protocol with corifollitropin alfa followed by hMG (named as *simplified long protocol*). Clinical outcomes were compared between groups and between the first/second cycle of each group.

**Results:**

Clinical outcomes were similar between groups. There were no differences in the number of oocytes [7(5-11.75) versus 7(5-10), *p*=0.802], clinical pregnancy (19.3% *versus* 18.2%, *p*=0.858) and live birth rates (18.2% *versus* 15.2%, *p*=0.619). However, baseline characteristics were different, decoding a poor prognosis among women in group 2. Both groups (1 and 2) had significantly higher number of oocytes, pregnancy, and live birth rates in the second cycle. In group 2, there was a higher rate of embryo transfer (56.1% *versus* 27.3%, *p*<0.001). In group 1, despite the similar rate of embryo transfer, there was a higher positive hCG (23.9% *versus* 8.0%, *p*=0.004).

**Conclusions:**

Both simplified long protocol and GnRH antagonist protocol are suitable for low responders. The best second cycle clinical outcomes experienced in a population with worse prognosis (group 2) suggests that the simplified long protocol may be a better option, although prospective well-conducted studies must explore this hypothesis.

## INTRODUCTION

The management of patients with impaired response to ovarian stimulation is still one of the biggest challenges regarding assisted reproduction treatments. The extensive literature published on this subject was being unfruitful due to lack of consensus in the definition of this population of patients, hampering the interpretation of the studies and the search for an adequate ovarian stimulation protocol.

The publication of the Bologna criteria in 2011 (Ferraretti *et al.*, 2011) was an important step to overcome this barrier, allowing to compare different ovarian stimulation protocols in a well-defined population. Poor or low responders are defined by the presence of at least two of the following three conditions: 1) advanced maternal aged (^3^ 40 years old) or any other risk factor for poor ovarian response; 2) a previous poor ovarian response (< 4 oocytes with a conventional stimulation protocol); 3) an abnormal ovarian reserve test [antral follicle count (AFC) < 5-7 follicles or Anti-Mullerian Hormone (AMH) < 0.5-1.1 ng/mL].

According to the meta-analysis from Lambalk *et al.* (2017), there are no differences in pregnancy and live birth rates when comparing the Gonadotropin Releasing Hormone (GnRH) antagonist protocol with the GnRH agonist protocol in low responders. Currently, the European Society of Human Reproduction and Embryology (ESHRE) Guideline Group on Ovarian Stimulation (Ovarian Stimulation TEGGO *et al.*, 2020) equally recommends GnRH antagonist and GnRH agonist protocols for low responders.

However, a more recent meta-analysis suggests that GnRH agonist may be a better option for the management of low responders. In their study, Papamentzelopoulou *et al.* (2021) have only included studies in which poor responders were defined by the Bologna criteria and reported a lower cancellation rate (OR=1.268 > 1, 95% CI 1.007, 1.598) and a higher clinical pregnancy rate (OR=0.748 < 1, 95% CI 0.588, 0.952) in the GnRH agonist in comparison with the GnRH antagonist protocol.

Despite the same pharmacodynamic effect as recombinant Follicle Stimulating Hormone (rFSH), because it interacts only with FSH-receptor, corifollitropin alfa has emerged as an alternative gonadotropin for poor responders due to its pharmacological characteristics and results from clinical studies. Corifollitropin alfa is a recombinant dimeric glycoprotein obtained by the fusion of rFSH with the carboxy terminal peptide of the b subunit of hCG, which gives this molecule a longer and sustained half-life that allows the induction and maintenance of multifollicular growth during 7 days with a single injection, documented in both preclinical (Verbost *et al.*, 2011) and clinical studies (Corifollitropin Alfa Dose finding Study Group, 2008; Devroey *et al.*, 2004). Also, corifollitropin provides a more rapid achievement and higher levels of FSH in the first three days in comparison to daily rFSH, which peaks on day 3 to 5 (Corifollitropin alfa Ensure Study Group, 2010; Devroey *et al*., 2004; Fauser *et al.*, 2010).

The efficacy and safety of corifollitropin alfa have been demonstrated in several clinical studies (Boostanfar *et al.*, 2015; Corifollitropin alfa Ensure Study Group, 2010; Devroey *et al.*, 2009; Devroey *et al*., 2004; Polyzos *et al.*, 2013; Pouwer *et al.*, 2015) showing similar rates of ongoing pregnancy, with the advantage of eliminating the inconvenience and discomfort of repeated injections. Although these studies have been conducted in normal responders, the follicular response with corifollitropin resulted in a higher number of oocytes retrieved (Corifollitropin alfa Ensure Study Group, 2010; Devroey *et al*., 2009), which explains the interest of using this gonadotropin in poor responders, either with daily rFSH or in association with human Menopausal Gonadotropin (hMG), adding the potential benefit of LH activity in this population (Alviggi *et al.*, 2018).

Nonetheless, RCTs comparing corifollitropin with other gonadotropins (rFSH and hMG) in a GnRH antagonist protocol in poor responders have shown no differences in the number of oocytes retrieved, pregnancy and live birth rates (Cozzolino *et al.*, 2019; Kolibianakis *et al.*, 2015; Taronger *et al.*, 2018).

In the abovementioned studies, poor responder patients were selected according to the Bologna criteria. Despite the general acceptance of this criteria by the scientific community, experts in this field pointed out the need to further classify this heterogeneous population in different groups, according to the degree of low prognosis, to optimize the clinical strategy.

In 2016, the Poseidon group (Poseidon Group *et al*., 2016) suggested the stratification of women into four groups, integrating the concept of a low qualitative ovarian reserve (age and risk of aneuploidy) with low quantitative ovarian reserve (according to ovarian biomarkers) and impaired response to ovarian stimulation. Groups 1 (young) and 2 (older) correspond to women with a normal ovarian reserve and an unexpected low or suboptimal response to ovarian stimulation. Groups 3 (young) and 4 (older) correspond to women with a low ovarian reserve, based on AFC and AMH.

Therefore, we hypothesized that a long GnRH agonist protocol with corifollitropin alfa followed by hMG (named as long simplified protocol) could be better for women with suboptimal response (Poseidon groups 1 and 2), despite an apparently normal ovarian reserve (who do not fulfil Bologna criteria).

For that, we retrospectively compared the outcomes of a second ovarian stimulation cycle with two different protocols (GnRH antagonist *versus* long GnRH agonist with corifollitropin alfa followed by hMG) in patients with a normal ovarian reserve and a previous low response to antagonist GnRH protocol (Poseidon groups 1 and 2).

## MATERIALS AND METHODS

An institutional review board approval was obtained to perform the study from the Ethical Committee of a Terciary University Hospital - Centro Hospitalar e Universitário de Coimbra (OBS.SF.044-2022).

### Study design and participants

We have conducted a retrospective cohort study at the Reproductive Medicine Unit of a University Hospital between October 2013 and October 2021.

Women/couples who met all the inclusion criteria were eligible to be included in the study.

The inclusion criteria were:

- Suboptimal ovarian response (defined as < 9 oocytes obtained) or a cancelled cycle after a GnRH antagonist protocol using rFSH/hMG;
- Normal ovarian reserve (defined as AFC > 5);
- Second ovarian stimulation protocol with a GnRH antagonist protocol using rFSH/hMG or a long GnRH agonist protocol using a single dose of triptorelin 3.75mg and corifollitropin alfa followed by hMG.

Patients with an ongoing pregnancy (after the first cycle) or with polycystic ovarian syndrome (PCOS) were excluded.

According to the second ovarian stimulation protocol, patients were classified in:

1. **Group 1**, submitted to a second cycle with a **GnRH antagonist protocol** using rFSH/hMG
2. **Group 2,** submitted to a long GnRH agonist protocol using a single dose of triptorelin 3.75 mg and corifollitropin alfa followed by hMG - named as ***simplified long protocol***.

This was a retrospective study, and a selection bias (indication bias) was promptly evident after comparing the first cycle of both groups, which had different prognostic features. Patients from group 2 were older, had a lower ovarian reserve (based on AFC and AMH) and poor outcomes in the first cycle (fewer oocytes and embryos). This was expected, since we usually offer the simplified long protocol to women with a poor prognosis/response.

Clinicians usually optimize the second ovarian stimulation protocol according to the previous response. Therefore, we also compared the outcomes of the **first** and **second cycle** according to each group (*i.e.,* in the same women/couple).

### Ovarian stimulation protocols, ovulation triggering and luteal phase support

#### GnRH antagonist protocol

On day 2 or 3 of the menstrual cycle, daily subcutaneous (SC) injections of rFSH or hMG were given up to the day of human chorionic gonadotropin (hCG) administration, when at least three leading follicles reached a mean diameter of 17 mm. Doses of rFSH/hMG ranged from 150 to 300 IU/day depending on the women’s age, body mass index (BMI), AFC and AMH. Administration of a daily dose of 0.25 mg of gonadotropin releasing hormone antagonist (GnRHa) (Cetrotide^®^, Merck, Netherlands; or Orgalutran^®^, Organon, Netherlands) was initiated when the larger follicle reached a mean diameter of 14 mm. Transvaginal oocyte retrieval was schedule to 36 hours after choriogonadotropin alfa (α-hCG) administration.

Luteal phase support with progesterone was initiated on the day of the oocyte retrieval with 200 mg of micronized intravaginal progesterone every 8 hours (Progeffik^®^, EFIKK, France).

#### Simplified long protocol

In the long agonist protocol, pituitary downregulation was induced by a single intramuscular (IM) administration of triptorelin 3.75 mg (Decapeptyl^®^, Ferring, Germany) in the midluteal phase (day 21) of the pretreatment cycle. After two weeks, downregulation was assessed by transvaginal ultrasound and patients start ovarian stimulation.

A single SC injection of corifollitropin alfa (Elonva^®^, MSD, Oss, Netherlands) was administered at stimulation day 1 (SD1). The dose of corifollitropin alfa was 100 mg for patients with a bodyweight £ 60 Kg and 150 mg for patients with a bodyweight > 60 Kg.

From SD8 onwards treatment continued with a daily SC dose of HP-hMG (Menopur^®^, Ferring, Germany), 225 IU/day up to the day of hCG administration, when at least three leading follicles reached a mean diameter of 17 mm. Transvaginal oocyte retrieval was scheduled to 36 hours after α-hCG administration.

Luteal phase support with estradiol and progesterone was initiated on the day of oocyte retrieval with 2 mg of oral estradiol (Zumenon^®^, Abbott Biologicals, Netherlands) and 200 mg of micronized intravaginal progesterone (Progeffik^®^, EFIKK, France) every 8 hours.

**Figure 1:**
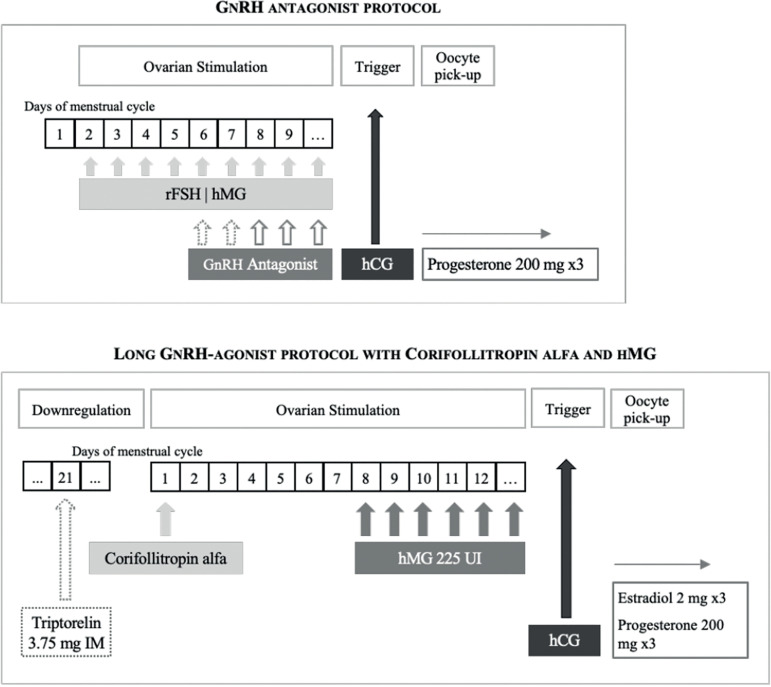
Ovarian stimulation protocol schemas. rFSH: recombinante Follicle Stimulating Hormone; hMG: human Menopausal Gonadotropin; GnRH: Gonadotropin Releasing Hormone; hCG: choriogonadotropin; IM: intramuscular.

A schematic description of both ovarian stimulation protocols is provided in Figure 1.

Fertilization was assessed after 18 hours and fertilization rates were calculated. Embryo cleavage was assessed every 24 hours thereafter and transfer was performed 3 or 5 days after oocyte retrieval. Fourteen days after oocyte retrieval a quantitative serum value of β-hCG was obtained and a transvaginal ultrasound was scheduled one week later in case of a positive result.

### Outcomes

The outcome measures were the number of oocytes collected and mature oocytes, the number of cancelled cycles, embryos obtained, positive hCG tests (positive β-hCG), clinical pregnancy (intrauterine gestational sac) and live births (fetus born alive beyond the 24^th^ week of gestation).

If oocyte retrieval was cancelled, patients were considered as having retrieved zero oocytes.

Clinical pregnancy was expressed per cycle started as well as per embryo transfer.

### Statistical analysis

For the first analysis, the outcomes of groups 1 and 2 were compared with the use of independent hypothesis tests. Continuous variables were compared with the use of T-test and categorical variables were compared with the Chi-squared or Fisher’s exact test, as appropriate.

For the second analysis, the outcomes of the second ovarian stimulation cycle were compared with the outcomes of the first cycle in the same women/couple using the paired T-test/Wilcoxon for numerical data and McNemar’s test for categorical data.

Data are presented as means (± standard deviation) or as median (interquartile range). Statistical analysis was performed with the support of IBM SPSS Statistics for Windows, (Version 20.0. IBM Corp, Armonk, NY, USA), with the level of significance fixed at 5%.

## RESULTS

One hundred and fifty-four women met the inclusion criteria, including the criteria to be classified in Poseidon groups 1 and 2. Those women were further classified according to their second ovarian stimulation protocol in Group 1 - GnRH antagonist protocol (n=88) or Group 2 - simplified long protocol (n=66).

### Baseline characteristics according to study group (group 1 *versus* group 2)

Baseline characteristics of patients and clinical outcomes from the previous cycle (first cycle) are shown in Table 1.

**Table 1:** Baseline characteristics and previous clinical outcomes (from the first cycle with GnRH antagonist protocol) in the group 1 (submitted to second GnRH antagonist protocol) *versus* group 2 (submitted to long GnRH agonist protocol with corifollitropin alfa followed by hMG - *simplified long protocol*.

	Group 1 GnRH antagonist	Group 2 Simplified long protocol	*p*-value
Number of patients/cycles, n	88	66	
Age (years), mean (SD)	34.6 (3.4)	35.6 (3.0)	0.076
BMI (kg/m^2^), mean (SD)	23.9 (4.3)	23.7 (3.6)	0.710
AMH (ng/mL), mean (SD)	2.27 (1.01)	1.65 (0.91)	<0.001
AFC, median (IQR)	10 (8-15)	9 (7-12.25)	0.014
Primary infertility cause, n (%) Male Tubal Endometriosis Idiopathic Other	27 (30.7) 22 (25) 4 (4.5) 31 (35.2) 4 (4.5)	18 (27.3) 22 (33.3) 9 (13.6) 13 (19.7) 4 (6.1)	0.645 0.257 0.045 0.035 0.675
Poseidon *subgroup a*, n (%)	13 (14.8)	18 (27.3)	0.056
Days of stimulation, median (IQR)	9 (8-10)	9 (8-10)	0.439
Number of oocytes retrieved, median (IQR)	6 (4-7)	5 (3-6)	0.015
Number of metaphase II oocytes, median (IQR)	4 (3-5.75)	3 (1-4)	<0.001
Embryos obtained, median (IQR)	1 (0-2)	0 (0-1)	<0.001
Cycle cancellation, *n* (%)	1 (1.1)	3 (4.5)	0.314
Embryo transfer, *n* (%)	52 (59.1)	18 (27.3)	<0.001
Embryos cryopreserved, median (min-max)	0 (0-3)	0 (0-1)	0.043
Positive hCG per cycle, *n* (%)	7 (8.0)	7 (10.6)	0.571
Clinical pregnancy per cycle, *n* (%)	5 (5.7)	1 (1.5)	0.186
Live birth per cycle, *n* (%)	0 (0)	0 (0)	---

n: number; IQR: interquartile range; SD: standard deviation.

As mentioned, ovarian reserve biomarkers were significantly different between the two groups. Group 2 had a lower AMH [1.65 (± 0.91) *versus* 2.27 (± 1.01), *p*<0.001] and a lower AFC [9 (7-12.25) *versus* 10 (8-15), *p*=0.014]. More women in group 2 had been diagnosed with endometriosis (13.6% *versus* 4.5%, *p*=0.045).

The median number (min-max) of cryopreserved embryos was 0 (0-3) in group 1 and 0 (0-1) in group 2, which was significantly different (*p*=0.043). Also, more women were classified in the Poseidon subgroup a (obtained less than 4 oocytes in the first cycle) (27.3% *versus* 14.8%, *p*=0.056) and the mean age of group 2 was higher (35.6±3.0 *versus* 34.6±3.4, *p*=0.076), although those differences did not reach statistical significance.

### Comparison of clinical outcomes according to study group (group 1 *versus* group 2)

Clinical outcomes are shown in Table 2.

**Table 2:** Clinical outcomes in the GnRH antagonist protocol (group 1) versus long GnRH agonist protocol with corifollitropin alfa followed by hMG - simplified long protocol (group 2).

	Group 1 GnRH antagonist	Group 2 Simplified long protocol	*p*-value
Interval between first and second cycle, days (SD)	312 (137)	289 (149)	0.355
Days of stimulation, median (IQR)	9 (8-10)	12 (11-13)	<0.001
Number of oocytes retrieved, median (IQR)	7 (5-11.75)	7 (5-10)	0.802
Number of metaphase II oocytes, median (IQR)	4.5 (3-7)	4 (3-7)	0.915
Embryos obtained, median (IQR)	1 (0-2)	1 (0-2)	0.884
Cycle cancellation, *n* (%)	2 (2.3)	1 (1.5)	1.000
Embryo transfer, *n* (%)	53 (60.2)	37 (56.1)	0.604
Embryos cryopreserved, median (min-max)	0 (0-7)	0 (0-6)	0.458
Positive hCG per cycle, *n* (%)	21 (23.9)	13 (19.7)	0.537
Clinical pregnancy per cycle, *n* (%)	17 (19.3)	12 (18.2)	0.858
Clinical pregnancy per transfer, *n* (%)	17 (29.8)	15 (35.7)	0.536
Live birth per cycle, *n* (%)	16 (18.2)	10 (15.2)	0.619

The interval between the first and the second cycles were 312±137 and 289±149 days for group 1 and group 2, respectively. The difference between groups was 23 days.

As expected, a longer duration of stimulation was observed in the group undergoing a long GnRH protocol [12 days (11-13) *versus* 9 days (8-10), *p*<0.001].

The number of oocytes, the clinical pregnancy and the live-birth rates were not statistically different between groups. All the other IVF outcomes evaluated were also comparable between groups.

### Comparison of clinical outcomes between cycles (first *versus* second cycle of each group)

The comparison between the first and second ovarian stimulation cycles in each group are shown in Tables 3A and 3B.

**Table 3A:** Comparison of the clinical outcomes between the first *versus* second cycle of each ovarian stimulation protocol group.

Outcomes	Group 1 n=88		95% CI of the difference between medians	*p*-value	Group 2 n=66		95% CI of the difference between medians	*p*-value
	First cycle GnRH antagonist	Second cycle GnRH antagonist			First cycle GnRH antagonist	Second cycle Simplified long protocol		
Days of stimulation, median (IQR)	9 (8-10)	9 (8-10)	0 to +0.5	0.359	9 (8-10)	12 (11-13)	+2 to +3	<0.001
Number of oocytes retrieved, median (IQR)	6 (4-7)	7 (5-11.75)	+1 to +3	<0.001	5 (3-6)	7 (5-10)	+2 to +4.5	<0.001
Number of metaphase II oocytes, median (IQR)	4 (3-5.75)	4.5 (3-7)	0 to +1.5	0.008	3 (1-4)	4 (3-7)	+1.5 to +3	<0.001
Embryos obtained, median (IQR)	1 (0-2)	1 (0-2)	0 to +0.5	0.342	0 (0-1)	1 (0-2)	+0.5 to +1	<0.001

n: number; IQR: interquartile range.

**Table 3B:** Comparison of the clinical outcomes between the first (same protocol - GnRH antagonist) versus second cycle (different protocols) of each ovarian stimulation protocol group.

Outcomes	Group 1 n=88		*p*-value	Group 2 n=66		*p*-value
	First cycle GnRH antagonist	Second cycle GnRH antagonist		First cycle GnRH antagonist	Second cycle Simplified long protocol	
Cycle cancellation, *n* (%)	1 (1.1)	2 (2.3)	0.564	3 (4.5)	1 (1.5)	0.317
Embryo transfer, *n* (%)	52 (59.1)	53 (60.2)	0.869	18 (27.3)	36 (56.1)	<0.001
Positive hCG per cycle, *n* (%)	7 (8.0)	21 (23.9)	0.004	7 (10.6)	12 (19.7)	0.157
Clinical pregnancy per cycle, *n* (%)	5 (5.7)	17 (19.3)	0.005	1 (1.5)	11 (18.2)	0.002
Live birth per cycle, *n* (%)	0 (0)	16 (18.2)	<0.001	0 (0)	10 (15.2)	<0.001

n: number.

Both groups (1 and 2) had significantly higher number of oocytes and mature oocytes and higher pregnancy and live birth rates in the second cycle.

The 95% confidence interval (CI) of the difference between medians in the number of oocytes retrieved was +1 to +3 in group 1 and +2 to +4.5 in the group 2 and in the number of metaphase II oocytes was 0 to +1.5 in group 1 and +1.5 to +3 in the group 2.

In group 1, the number of embryos obtained, and the rate of embryo transfer was similar between first and second cycle, although a higher positive hCG was observed (23.9% *versus* 8.0%, *p*=0.004).

In group 2, there was a significant difference between the number of embryos obtained in the second cycle (1 *versus* 0, *p*<0.001), as well as the rate of embryo transfer (56.1% *versus* 27.3%, *p*<0.001).

Both groups had higher clinical pregnancy and live birth rates in the second cycle.

## DISCUSSION

Low responders had similar clinical outcomes when submitted to a second ovarian stimulation with simplified long protocol (group 2) versus GnRH antagonist protocol (group 1). Accordingly, both protocols seem to be suitable for low responders. However, since group 2 had a worse prognosis than group 1, we next compared the first and second cycle of each group. Group 2 had more oocytes and more embryos to transfer, suggesting a more adequate ovarian response in the second ovarian stimulation protocol.

Our hypothesis of having better outcomes with corifollitropin alfa was based on the potential benefit of having a higher number of oocytes due to the rapid increase and higher levels of FSH (Fauser *et al*., 2010) that could recruit more follicles. Indeed, in normoresponders, the follicular response with corifollitropin resulted in a higher number of oocytes retrieved (Corifollitropin alfa Ensure Study Group, 2010; Devroey *et al*., 2009). On the other hand, studies conducted in poor responders, comparing corifollitropin alfa with other gonadotropins, reported no differences in the number of oocytes collected, as well as pregnancy and live birth rates (Drakopoulos *et al.*, 2017; Kolibianakis *et al*., 2015; Taronger *et al*., 2018). In our study, ovarian stimulation with corifollitropin alfa was not superior to rFSH/hMG in patients with a suboptimal previous response, which is in accordance with other published studies (Kolibianakis *et al*., 2015), even when corifollitropin is followed by hMG (Drakopoulos *et al*., 2017).

We have also hypothesized that the long GnRH agonist could be a better option for poor responders, has suggested by others (Papamentzelopoulou *et al*., 2021). In their meta-analysis, Papamentzelopoulou *et al*. (2021), reported a lower cancellation rate (OR=1.268 > 1, 95% CI 1.007, 1.598) and a higher clinical pregnancy rate (OR=0.748 < 1, 95% CI 0.588, 0.952) in the GnRH agonist in comparison with the GnRH antagonist protocol.

The strategy to suppress endogenous FSH in the luteal phase has been adopted in low responders with the aim of inhibiting the premature recruitment and selection of follicles, allowing for a synchronous follicular growth. This strategy has also been achieved by using the “delayed start antagonist protocol”, with higher pregnancy rates in comparison to the antagonist protocol (Cozzolino *et al.*, 2020; Yang *et al.*, 2021).

Comparison of corifollitropin using a short *versus* long agonist protocol in poor responders was performed by Polyzos *et al.* (2015). Their prospective study demonstrated similar results, with no differences in the number of oocytes retrieved nor in pregnancy and live birth rates. Fusi *et al.* (2020) conducted an RCT comparing three different protocols in poor responders: 1) GnRH antagonist without corifollitropin, 2) GnRH antagonist with corifollitropin and 3) Long GnRH agonist with corifollitropin. The authors reported a higher number of oocytes retrieved and a lower cancellation rate in the corifollitropin groups (with both agonist and antagonist protocols).

Conversely, in our population of patients with a previous suboptimal response (Poseidon groups 1 and 2), either long GnRH agonist protocol with corifollitropin and GnRH antagonist resulted in similar clinical outcomes. There were two cancelled cycles in the GnRH antagonist (2.3%) and one in the long GnRH agonist with corifollitropin (1.5%), although no conclusions can be made regarding cycle cancellation rate due to the small population size.

As mentioned, group 2 had a poor prognosis in relation to group 1, comprising a population of older women with a lower ovarian reserve, more cases of endometriosis as primary cause of infertility and worse outcomes in the first cycle (fewer oocytes and embryos).

Regarding the comparison of the first and second cycle in each group, we could notice that group 1 (GnRH antagonist protocol) had a higher positive hCG rate in the second cycle, with similar number of embryos obtained and embryo transfer rate, suggesting a higher embryo quality in the second cycle. On the other hand, group 2 had more oocytes and more embryos to transfer, suggesting a more adequate ovarian response in the second ovarian stimulation protocol.

Therefore, low responders may benefit from a long GnRH agonist protocol with corifollitropin alfa followed by hMG. This hypothesis should be explored in an RCT, eliminating the selection bias of our retrospective study. As mentioned, when comparing the population of groups 1 and 2, an indication bias was evident from the poor prognostic features of women selected to undergo an ovarian stimulation protocol with simplified long protocol (group 2). The duration of the study was another limitation, considering that laboratory techniques may have changed during the study period.

The statistical power of our study was 80% to detect a difference of 14% in the clinical pregnancy rate, based on the clinical pregnancy rate of poor responders with the GnRH antagonist protocol and the simplified long protocol, which were 4% and 19%, respectively. Therefore, the study was powered to detect differences in clinical pregnancy rate, which validate our results in terms of sample size, although the methodology and bias of our study still limits the recommendation of one protocol for women with a suboptimal ovarian response.

Notwithstanding, the simplified long protocol has the advantage of being more friendly, regarding the inconvenience and discomfort of repeated injections. In addition, according to Cruz *et al*. (2017) corifollitropin alfa is less cost-effective than other gonadotropins (recombinant FSH and hMG).

The novelty of our study was the selection of patients. Protocols with corifollitropin followed by hMG have already been explored in patients who fulfilled Bologna criteria but not in patients with a suboptimal response to a previous ovarian stimulation cycle (Poseidon groups 1 and 2), despite the absence of any other criteria for poor response. Kolibianakis *et al*. (2015) have also selected patients with a previous low response, but they have included women who met other Bologna criteria for poor response, including more than 40 years old and a low ovarian reserve.

In conclusion, ovarian stimulation protocols using GnRH antagonist or long GnRH agonist with corifollitropin alfa followed by hMG (simplified long protocol) are both suitable to use in the second cycle of patients with a previous unexpected suboptimal response. The best second cycle clinical outcomes experienced by a poor prognosis population (group 2) suggests that the long GnRH agonist with corifollitropin alfa followed by hMG may be a better option for low responders, although prospective well-conducted studies must explore this hypothesis.
